# Immunotherapeutic Strategies for Intra-cranial Metastatic Melanoma - a Meta-analysis and Systematic Review

**DOI:** 10.7150/jca.93306

**Published:** 2024-05-05

**Authors:** Hiu Kwan Carolyn Tang, Ankit Rao, Christina Peters, Tanvi Ambulkar, Michael FX Ho, Bo Wang, Poulam Patel

**Affiliations:** 1 Department of Oncology, Nottingham City Hospital, Hucknall Road, Nottingham, NG5 1PB, United Kingdom.; 2 Trinity Hall, University of Cambridge, Cambridge, CB2 1TJ, United Kingdom.

**Keywords:** immune checkpoint inhibitors, immunotherapy, intra-cranial, malignant melanoma

## Abstract

Immune-activating anti-CTLA4 and anti-PD1 monoclonal antibodies (alone or in combination) are being used to treat advanced melanoma patients and can lead to durable remissions, and long-term overall survival may be achieved in between 50-60% of patients. Although intracranial metastases are very common in melanoma (about 50-75% of all patients with advanced disease), most of the pivotal prospective clinical trials exclude patients with intra-cranial metastases, certainly if their lesions are symptomatic and steroid-requiring and the degree of sensitivity of intra-cranial melanoma to immunotherapy remains uncertain, and requires further investigation especially in view of the demonstrable activity of RAF-MEK inhibitors in this clinical setting and the emergence of stereotactic radiotherapy. Our study aimed to evaluate the efficacy and toxicity of immunotherapy against advanced melanoma patients with brain metastases. In terms of comparative studies, only retrospective analyses could be identified. Based on 3 retrospective studies, treatment of patients with melanoma brain metastases with immunotherapeutic approaches improves overall survival substantially compared with supportive measures alone (no active anticancer treatment). The efficacy of targeted therapy appeared to be comparable to that of immune therapy in terms of overall survival, based on a small number of patients. The combination of concurrent radiation therapy to the brain and systemic immunotherapy led to improved overall survival compared to radiotherapy alone, suggesting potential synergism between the approaches, and combination treatment could be delivered safely. Our review supports the use of immunotherapeutic strategies for these patients although treatment efficacy appears to be lower for symptomatic lesions. In view of the extremely high efficacy of stereotactic radiotherapy approaches in the brain, understanding the interaction between radiotherapy and immunotherapy is vital and should be an area of active investigation.

## Introduction

The incidence of cutaneous malignant melanoma is rising rapidly and it is an increasing healthcare burden 1. Of all malignancies, melanoma (cutaneous or mucosal) has a very high propensity for central nervous system (CNS) dissemination, and CNS metastases are a common and lethal complication of malignant melanoma. Based on a study of people diagnosed between 1986 and 2004, when effective systemic therapy was unavailable, they have generally had a very poor prognosis of five months 2. Despite the anatomical proximity of the eye and brain, brain metastases are rare in uveal melanoma 3. About half of people with metastatic melanoma will develop CNS involvement at some point in their disease trajectory 4. CNS involvement may manifest as parenchymal brain metastases that are often associated with haemorrhage, or more diffuse lepto-meningeal encasement of the neuraxis. Whole brain radiotherapy, potentially applicable to patients, has marginal efficacy for CNS metastatic melanoma and is associated with cognitive deterioration 5. A small proportion of people with isolated (usually solitary) CNS lesions may achieve long-term survival with neurosurgical excision. CNS metastases often lead to progressive neurological deficits, functional impairment, disability, marked deterioration in quality of life, and seizure activity.

Although neurological symptoms from melanoma CNS metastases can be controlled by corticosteroids through reduction of peri-lesional oedema, the immunosuppressive effect of corticosteroids is likely to nullify the therapeutic effect of immune checkpoint inhibitors, which are now a key component of the therapeutic armamentarium for melanoma 6. This has led to the increasing use of neuroimaging, particularly in the form of contrast-enhanced magnetic resonance imaging (MRI), to screen for asymptomatic brain metastases to give systemic therapies and stereotactic radiosurgery the best chance of being effective. In the presence of the B-Raf proto-oncogene, serine/threonine kinase (BRAF) V600 mutation (40% to 50% of cutaneous melanomas), RAF- and MEK-targeted therapies can be effective for CNS metastatic melanoma in the short to medium term, with a high probability of symptomatic improvement even in people dependent on steroids albeit with a very low probability of long-term disease control and survival 7.

Immunotherapy is a unique form of cancer treatment that encourages the body's immune system to recognise and attack the cancer cells rather than having any direct effect on the cancer itself, unlike with chemotherapy and radiotherapy. Melanoma skin cancer has many genetic changes due to the effect of ultraviolet light damage, which makes it quite visible to the body's immune system, allowing immune cells to enter the brain to treat melanoma that has spread to this part of the body.

Melanoma is considered an immunogenic cancer, based on several observations including: 1) the frequency of spontaneous regressions of metastatic melanoma (often accompanied by vitiligo); 2) the prognostic significance of the density of tumour-infiltrating-lymphocytes in the primary lesions; and 3) the efficacy of non-specific immune stimulation with interleukin-2 (IL-2) for advanced disease. Systemic therapy options for metastatic melanoma have expanded recently with the development of immune checkpoint inhibitors (monoclonal antibodies targeted against cytotoxic T-lymphocyte associated protein 4 (CTLA4) and programmed cell death 1 (PD-1), which has transformed the outlook and prognosis for patients, with long-term survival now a realistic goal in the majority 8. It must be noted that the pivotal clinical trials only included patients in a good general condition with World Health Organization (WHO) performance status of 0 to 1. In contrast, median overall survival with conventional cytotoxic chemotherapy (such as dacarbazine) was approximately six to nine months 9. Immune checkpoint blockade attenuates inhibitory signals to T-cells, and shifts the balance away from tumour-driven T-cell suppression towards sustained activation, proliferation, and cytotoxic function of T-cells as part of an effective antitumour response 10. The National Institute for Health and Care Excellence (NICE) approved ipilimumab (anti-CTLA4) for the treatment of metastatic melanoma in 2012 11, followed by pembrolizumab and nivolumab, which target the PD-1 axis. Ipilimumab is approved in combination with nivolumab, with a favourable outcome compared with ipilimumab monotherapy, although the superiority of combination therapy over anti-PD-1 monotherapy is less when tumour PD-L1 expression is greater than 5% 12,13. Anti-PD-1 monotherapy is better tolerated than chemotherapy, and has demonstrated superior progression-free survival compared with chemotherapy, with lower rates of severe treatment-related toxicity 14,15. Although the contemporary focus is on immune checkpoint blockade, cellular vaccination approaches (e.g. using dendritic cells 16), as well as cytokine therapy (e.g. intravenous high-dose IL-2), adoptive T-cell therapy 17, and intra-lesional viral immunotherapy (e.g. talimogene laherparepvec 18), continue to have important roles in combating this disease. Combinations of different immunotherapeutic modalities (e.g. checkpoint blockade alongside antigen-specific vaccination, NCT04079166 19, and RAF-MEK-targeted therapy, NCT02902042 20, are being actively explored and may optimise treatment outcomes 21. Whilst not in routine clinical use currently, the composition and diversity of the faecal microbiome 22, and the tumoural somatic mutational load 23, may develop into predictive biomarkers for cancer immunotherapy, allowing people who are unlikely to benefit to be spared from unnecessary toxicities.

Conventionally, the brain has been considered to be an immune-privileged site due to the absence of lymphatic drainage and the blood-brain barrier 24. However, it has been shown that activated T-cells can traverse the blood-brain barrier, especially when disrupted by metastatic disease, thereby creating the novel possibility of treatment using immunotherapy 25. It has also been demonstrated that, using immunohistochemical analysis of melanoma brain metastases, three-quarters of melanoma CNS lesions exhibit tumour-infiltrating T-lymphocytes, and about half have detectable PD-L1 expression on tumour cells. This provides solid evidence that the host adaptive immune response can be active in the distinct micro-environment of the brain 26. In fact, the expression levels of the molecular target for immunotherapeutic drugs such as nivolumab and pembrolizumab - PD-L1 - are higher on intra-cranial compared with extra-cranial melanoma metastases 27. The efficacy of immunotherapy for CNS metastases may be enhanced by concurrent treatment with localised approaches such as stereotactic radiotherapy (which has overcome the resistance of melanoma to conventionally delivered radiation therapy 28) due to radiation-induced upregulation of major histocompatibility complex (MHC) class I molecules on tumour deposits making them visible to CD8 T-cells 29, and immunogenic cell death and priming of immune responses against tumour antigens 30. In terms of clinical evidence, early data from a single-arm study of 76 patients found that ipilimumab and nivolumab immunotherapy achieved a radiologic response (lasting at least three months) in brain metastases of 51%, with a response in 20% of those receiving nivolumab monotherapy in asymptomatic patients. However, in patients with neurologic symptoms, lepto-meningeal metastases, or prior local treatment (i.e. radiotherapy), nivolumab only achieved intracranial response in 6%, highlighting the challenges of this approach 31. Consensus guidelines (European Society for Medical Oncology (ESMO)) recommend the use of combination immunotherapy in patients with asymptomatic melanoma brain metastases.

There is a paucity of clinical evidence on which to base decision-making in this group of people, who have typically been excluded from prospective clinical trials, and thus their outlook has remained poor; this is clearly an area of unmet medical need. More than half of metastatic melanoma patients develop CNS metastases, emphasising the need for a new therapeutic approach. Although the level of evidence is limited, there are preliminary insights that immune checkpoint inhibitors are active in melanoma brain metastases. In particular, a small study restricted to patients with small (less than 2 cm) asymptomatic brain metastases found an intracranial response rate of 22% with pembrolizumab, including a response lasting 10 months 32. Ipilimumab and nivolumab combination immunotherapy achieved an intracranial response rate of 55% in patients with small (less than 3 cm) asymptomatic brain metastases, including 26% complete responses, and almost 60% of patients were free of intracranial progression at nine months 33. These studies did not include those with neurologic symptoms due to metastatic lesions or those with prior local treatment such as radiotherapy. It is also important to conduct this review to understand how immunotherapy and localised treatment approaches such as stereotactic radiotherapy (which is associated with high rates of local disease control 34) should be combined in an integrated, multimodality approach with a particular focus on whether there is any synergistic benefit and whether the two modalities can be delivered together without excessive toxicities (i.e. brain radio-necrosis).

Therefore, the aims of this study were to critically appraise the safety data of immunotherapy by identifying rates of toxicities focusing on neurologic adverse events, to compare the survival outcomes of immunotherapy including progression free survival, overall survival, and clinical response rate, and to appraise evidence-based treatments for intracranial metastatic melanoma.

## Materials and Methods

Randomised controlled trials (RCTs) and both retrospective and prospective cohort studies were reviewed. Due to the extreme paucity of prospective, randomised trials for patients with melanoma brain metastases, in particular those with symptomatic lesions, retrospective cohort studies were permitted for inclusion in the review and meta-analysis. Some of these retrospective studies may have uses propensity score matching. We have not restricted studies to ones that use statistical adjustment for baselines imbalances that may lead to selection and other biases, but we have interpreted the findings accordingly. We did not present results in core sections of the review if we deemed studies to be at high risk of bias such that their inclusion would present blatantly biased results.

Patients with stage IVd malignant melanoma with symptomatic or asymptomatic intracranial metastases aged 18 years of age or over (AJCC 8th edition staging, 2018 35) were studied. Patients with both BRAF mutant and BRAF wild-type melanoma were included for analysis. The primary melanoma sites could have been be cutaneous or mucosal (but not uveal). Any form of immunotherapy was eligible for inclusion, including combination of CTLA4 and PD-L1 immune checkpoint blockade or either as single agents, as well as cytokine-based (i.e. IL-2) and vaccination approaches and adoptive T-cell therapy. We included studies where Immunotherapy could have been the single modality of therapy or given in combination with localised treatment approaches such as stereotactic radiosurgery.

We focused on the following outcomes measures - overall survival (i.e. survival from when participants were enrolled in the study to death from any cause) as well as the frequency and severity of neurological toxicities, focusing on symptoms of raised intracranial pressure, seizure activity, and intracerebral haemorrhage. The secondary outcomes were progression-free survival (both intracranial and extracranial, time from enrolment in study to radiological progression or death), quality of life, assessed by scales such as the Brain Symptom and Impact Questionnaire (BASIC) and intra-cranial objective response (i.e. greater than 30% reduction in target lesion dimension from baseline).

Relevant studies were identified using systematic searches of the electronic bibliographical databases - CENTRAL on the Cochrane Library, MEDLINE (Ovid), Embase (Ovid) and searches were from 1990 to the present time.

We searched Physician Data Query (www.nci.nih.gov) and the National Cancer Institute (www.cancer.gov/clinicaltrials) for ongoing trials. We also screened trial registers including the US National Institutes of Health Ongoing Trials Register ClinicalTrials.gov (clinicaltrials.gov/), metaRegister of Controlled Trials (www.controlled-trials.com/mrct/), and the EU Clinical Trials Register (www.clinicaltrialsregister.eu/ctrsearch/search). We checked the citation lists of included trials, key textbooks, and previous systematic reviews through handsearching, and contact experts in the field in the form of clinical colleagues to identify any further reports of trials. We also screened relevant conference abstracts (focusing on ESMO meetings, National Cancer Research Institute meeting, and Melanoma UK meetings) within the past five years.

**Figure 1:**
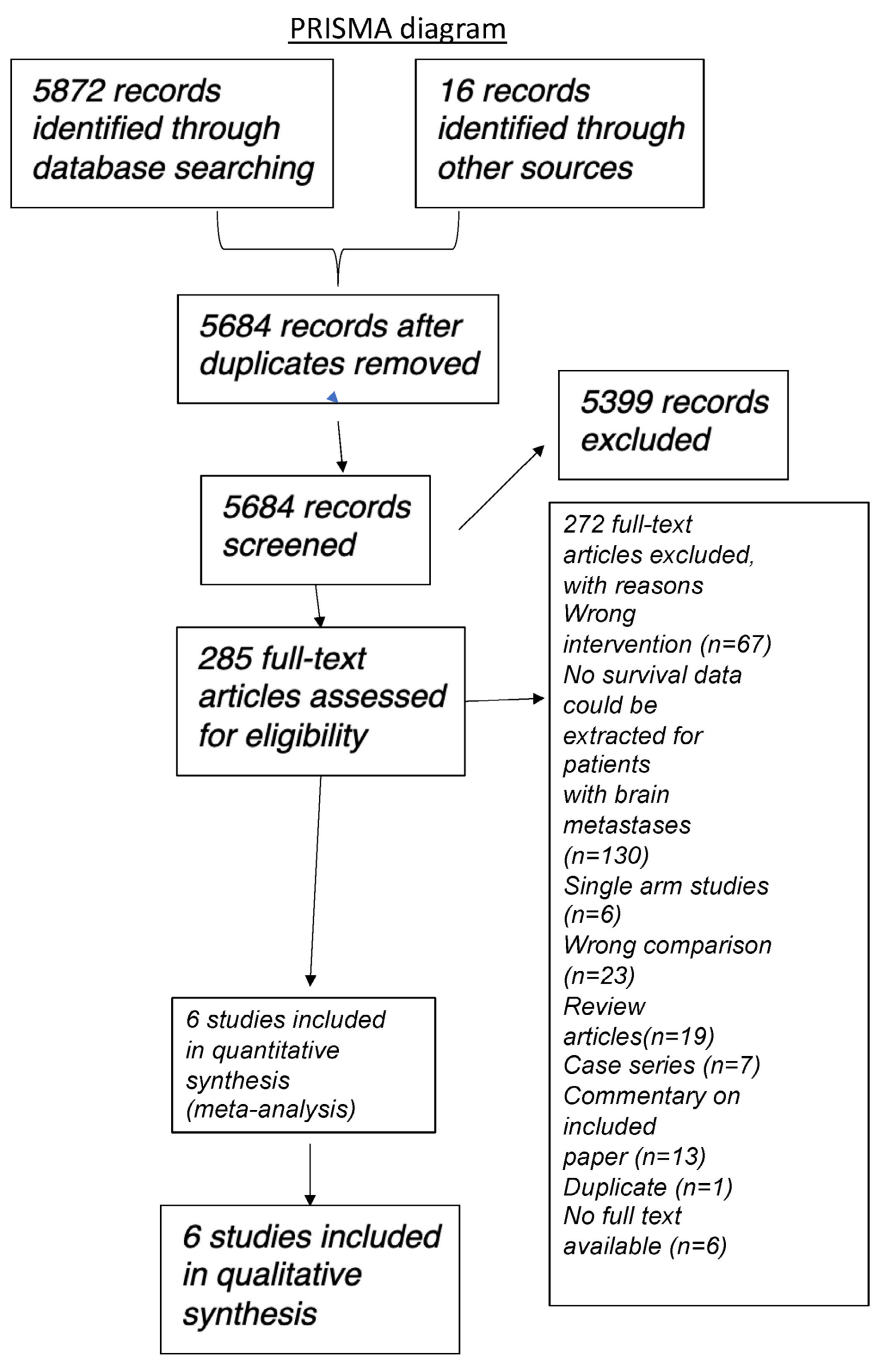
PRISMA diagram illustrating literature search strategy.

Three review authors, working independently, critically appraised the studies identified using the search strategy above for inclusion in the review. We screened the titles and abstracts for potentially relevant studies, and obtained the full-text articles of relevant studies for further assessment. When the article was not accessible online, steps were taken to contact the author to obtain the study article or the study outcome. If there is any difference in opinion, this was discussed through meetings and documented with further opinions sought from a fourth review author. We produced a PRISMA study flow diagram to demonstrate the papers that were searched for and selected (Figure 1).

Two review authors independently extracted relevant data from the included studies using a recognised data collection tool 36. This included abstracted data on characteristics of participants (inclusion criteria, age, stage, comorbidity, previous treatment, number enrolled in each arm/group) and immunotherapy intervention details (combination details, single agents, cytokine-based interventions, vaccination approaches, localised treatment approaches, etc.), risk of bias, duration of follow-up, outcomes, and deviations from protocol. We extracted data on the following outcome measures. For dichotomous outcomes (e.g. adverse events or deaths if it was not possible to use a hazard ratio), we extracted the number of participants in each treatment arm who experienced the outcome of interest and the number of participants assessed at endpoint, in order to estimate a risk ratio. For continuous outcomes (e.g. quality of life measures), we extracted the final value and standard deviation of the outcome of interest and the number of participants assessed at endpoint in each treatment arm at the end of follow-up, in order to estimate the mean difference between treatment arms and its standard error. For time-to-event (survival and disease progression) data, we will extract the log of the hazard ratio (log (HR)) and its standard error from trial reports; if these are not reported, we will attempt to estimate the log (HR) and its standard error using the methods of Parmar 37, or contact the trial authors. Where possible, all data extracted were relevant to an intention-to-treat analysis, in which participants are analysed in the groups to which they have been assigned. We noted the time points at which outcomes are collected and reported, and report unadjusted and adjusted results.

Two review authors (HKCT, AR) independently assessed the risk of bias in included studies using a recognised 'Risk of bias' tool 38. We assessed for selection bias, performance bias, attrition bias, detection bias, and reporting bias, and judge them either low risk, high risk, or unclear. The two review authors independently assessed the overall bias for each study and its potential impact on the study findings. If there was difference in opinion, this was discussed through a meeting and a third review author's opinion was sought.

We will present the overall certainty of the evidence for each outcome according to the GRADE approach, which takes into account issues not only related to internal validity (risk of bias, inconsistency, imprecision, publication bias), but also to external validity, such as directness of results 39,40. We will use the GRADE approach, to assess the certainty of the evidence related to each of the key outcome measures. We will create 'Summary of findings' tables using GRADEpro GDT software (Appendix 2) (GRADEpro GDT). We will use the GRADE checklist and GRADE Working Group definitions of the certainty of evidence 41. We will downgrade the evidence from 'high' certainty by one level for serious (or by two levels for very serious) concerns regarding each limitation, as follows. High-certainty: we are very confident that the true effect lies close to that of the estimate of the effect. Moderate-certainty: we are moderately confident in the effect estimate: the true effect is likely to be close to the estimate of the effect, but there is a possibility that it is substantially different. Low-certainty: our confidence in the effect estimate is limited: the true effect may be substantially different from the estimate of the effect. Very low-certainty: we have very little confidence in the effect estimate: the true effect is likely to be substantially different from the estimate of effect.

## Results and Discussion

We were only able to identify retrospective cohort clinical studies as there were no randomised (comparative) controlled trials available. In total, the initial literature search identified 5888 studies (5872 from databases and 16 from conference searching), and after 204 duplicates were removed, 5683 studies were screened via review of the abstract. Of these, 5398 were excluded based on title and/or abstract ineligibility. Therefore, 285 studies were selected for full text review and of these, 13 were selected for inclusion in the review. 4 prospective clinical trials (6 published articles) were identified but they were not included in the analysis as they were all single arm studies or with the wrong comparison (e.g. comparison between different immunotherapeutic modalities).

13 clinical studies met the inclusion criteria, and all were included in the quantitative synthesis and meta-analysis.

Diao *et al.* was a single-centre retrospective cohort-based analysis of 91 patients with melanoma brain metastases treated with brain stereotactic radiosurgery with or without ipilimumab monotherapy (3mg or 10mg/kg) 42. 91% of included patients had excellent performance status (Karnofsky performance score of at least 80) and the median brain metastasis size receiving SRS was small at 0.27cm^3^. Median overall survival was 15.1 months in those receiving ipilimumab and SRS and 7.8 months with radiotherapy alone. However, the rate of acute neurological toxicity with SRS and concurrent ipilimumab was 26% including events of cerebral oedema and haemorrhage.

Foppen *et al.* was one of rare studies reporting on outcomes for patients with lepto-meningeal dissemination of malignant melanoma (diagnosis based on MR imaging and/or CSF cytology) 43. This was a retrospective single-centre analysis of 39 patients treated in the Netherlands. As expected with lepto-meningeal metastases, median overall survival was low at 3 weeks in patients receiving no active anti-cancer treatment but was 17 weeks in those receiving radiotherapy, immunotherapy or molecularly-targeted therapy. Median overall survival was 4 weeks in those receiving radiotherapy alone, 6 weeks with ipilimumab (without radiotherapy) and 47 weeks with the combination of ipilimumab and radiotherapy.

Gabani *et al.* was a retrospective study, using the US National Cancer Database, of 1104 patients with melanoma brain metastases treated between 2011 and 2013 with cerebral radiotherapy with (n=192) or without immunotherapy (n=912) 44. After using propensity-score matching to correct for imbalances in baseline characteristics and prognostic factors, median overall survival was 11.1 months (8.9-13.4) for immunotherapy with radiotherapy and 6.2 months (5.6-6.8) for radiotherapy alone. Patients in this study predominantly received ipilimumab with only a small proportion receiving anti-PD-1 therapy.

Vosoughi *et al.* was a United States based retrospective cohort study, at a single-centre, of 79 patients with melanoma brain metastases treated with radiotherapy alone or systemic therapies including immunotherapy, BRAF targeted therapy or cytotoxic chemotherapy 4. 50% of patients had multiple brain metastases and 25% had a largest lesion diameter of 3cm or more. 46% of patients had neurological symptoms. Median overall survival was 15.4 months with RT alone, 38 months with anti-PD-1 containing immunotherapy (whether as monotherapy or alongside initial ipilimumab), 19.2 months with ipilimumab and 12.4 months with RAF (+-MEK) inhibitors.

Kaidar-Person *et al.* was a single-centre, retrospective cohort study of a total of 58 patients with melanoma brain metastases treated with radiation therapy alone (stereotactic) or in combination with immune checkpoint inhibitors 45. The patients were of excellent performance status with a median Karnofsky Performance Score of 90/100 and median age of 58 years. The GPA scores were similar in the two groups although more patients in the immunotherapy and RT group received subsequent RAF and MEK inhibitors compared with the RT alone group. 19 patients received ipilimumab monotherapy, 3 anti PD-1 monotherapy and 7 combined ipilimumab-nivolumab. Median diameter of the largest brain lesion was 15-20mm and the mean number of brain lesions treated with RT was 2. The majority of patients received immunotherapy after initial RT. 13% of patients in the RT and immunotherapy cohort developed brain radio-necrosis whilst this did not occur in the RT alone group. However, only 25% of cases of radio-necrosis were symptomatic. Cerebral haemorrhage was more frequent in the combination therapy group, but also occurred in those who received radiotherapy alone. Median overall survival was significantly longer in those receiving immunotherapy in addition to RT (15 versus 5.5 months).

Mangana *et al.* was a Swiss multi-centre retrospective cohort study of patients with Stage IV malignant melanoma with a subgroup of 61 patients with brain metastases present at the time of first diagnosis of metastatic disease 46. Patients received systemic therapy between 2008 and 2014. Median overall survival was 6.1 months with cytotoxic chemotherapy (n=18), 7.2 months with molecularly-targeted therapy (n=23), 10.9 months with immunotherapy (n=14) and 9.1 months with immunotherapy and targeted therapy (n=6). Patient numbers in this study are small and there is no detailed description of the nature of the brain metastases nor any data regarding concurrent localised treatment such as radiotherapy. In terms of the immunotherapy these patients received, 30% received anti-PD-1 monotherapy and 70% ipilimumab.

Silk *et al.* was a single-centre retrospective cohort study performed between 2005 and 2012 at the University of Michigan Cancer Centre, USA 47. The total study population comprised 70 patients and of these 33 also received immunotherapy with ipilimumab (monotherapy) and 37 patients received radiation therapy alone. In total, 37 patients received whole-brain radiotherapy and 33 patients received stereotactic radio-surgery. The vast majority of patients had a primary cutaneous melanoma (97%). Approximately half of patients had greater than 3 brain metastases. 45 patients were neurologically asymptomatic and 25 had neurological symptoms. More patients in the ipilimumab-RT group had received prior radiotherapy and far more patients in the ipilimumab-RT group received RAF+- MEK inhibitors than those in the RT alone group. Median overall survival, regardless of the radiotherapy modality received, was 5.3 months with RT alone and 18.3 months with RT and ipilimumab. This improvement in overall survival with ipilimumab was clearly driven by the patients received stereotactic RT since median overall survival was 5.3 months with whole-brain RT alone and 3.1 months with WBRT and ipilimumab. Brain radio-necrosis was only observed in the patients receiving RT and ipilimumab, however, rates of cerebral haemorrhage were lower in patients receiving ipilimumab. The improvement in overall survival with the addition of ipilimumab to RT in this study may be partially explained by the lower proportion of symptomatic patients in the RT+ipilimumab group, better performance status in this group and the fact that 39% of patients in this group received subsequent BRAF targeted therapy compared with 3% in the RT only group.

Patel *et al.* was a small, single-centre United States retrospective cohort study reporting on 54 patients with melanoma brain metastases treated with stereotactic radiotherapy with or without ipilimumab immunotherapy 48. The patients received stereotactic radiotherapy upfront (they had not received prior whole-brain radiotherapy) followed by the commencement of ipilimumab (3mg/kg every 3 weeks for 4 cycles) within 4 months of SRS. The radiotherapy dose ranged for 15 to 21 Gy depending on target lesion size. 60% of patients had multiple brain metastases. There was no difference in overall survival in the whole cohort depending on whether patients received ipilimumab in addition to radiotherapy. However, sub-group analysis found that for patients who received their first dose of ipilimumab within 14 days of radiotherapy, one and two year overall survival was 43.9% and 43.9% with SRS and ipilimumab compared with 38.5% and 25.7% with radiotherapy alone, suggesting that certainly at the 2 year landmark, the addition of ipilimumab improved survival. The chance of brain radio-necrosis (based on imaging) within 1 year of radiotherapy was higher in patients receiving additional ipilimumab (30% versus 20%), however, the rate of symptomatic brain radio-necrosis was identical in the two groups, suggesting that combined treatment can be safely delivered.

Ladwa *et al.* reported on a consecutive series of 154 patients, retrospectively analysed with melanoma brain metastases at two centres in Queensland, Australia from 2009 to 2016 49. This real-world retrospective analysis found that median overall survival was similar with no statistically significant difference according to whether or not the patients had received immunotherapy or not (9 months versus 8 months). However, despite overall similarities in overall survival, the use of immunotherapy was associated with an intracranial radiologic response rate of 34% with 12% complete responses. In fact, in BRAF mutant melanoma patients with brain metastases progressing after RAF +- MEK inhibitors, median overall survival was 7 months in those receiving immunotherapy compared with 2 months in those who did not, although this was not statistically significant.

Knisely *et al.* was a single-centre retrospective cohort analysis focusing on 77 patients with melanoma brain metastasis who received stereotactic radio-surgery for localised treatment 50. Two-thirds of patients were male and the median age was 61 years. 27 of 77 patients (35%) received systemic immunotherapy with ipilimumab alongside radiation therapy. Median overall survival was 21.3 months in those receiving SRS and ipilimumab and 4.9 months in those receiving SRS only. 2 year overall survival was 47% compared with 19% in the SRS and ipilimumab versus SRS only groups. In the combination therapy group, overall survival was identical in patients who receiving ipilimumab before or after SRS. Cases of symptomatic brain radio-necrosis were reported in this study, but the frequency was not described.

Forschner *et al.* was a German multi-centric retrospective cohort study of 105 patients with melanoma brain metastases receiving systemic therapy 51. Median overall survival was better at 14 months with RAF +- MEK inhibitors compared with 7 months for ipilimumab treated patients and those receiving cytotoxic chemotherapy had a median overall survival of 9 months. These findings likely reflect the high efficacy of BRAF targeted therapies even in the brain in the short to medium term and the lower biological activity of ipilimumab monotherapy.

Lang *et al.* was a small retrospective single-centre cohort study from The University of Mainz Cancer Centre in Germany of 22 patients with melanoma brain metastases treated with ipilimumab immunotherapy or vemurafenib (RAF inhibitor) monotherapy 52. Median overall survival for these patients with mainly large symptomatic brain metastases without options for stereotactic radiotherapy was generally poor and was 6 months for those with BRAF mutation treated with vemurafenib and 4 months in those treated with ipilimumab.

Iorgulescu was a retrospective United States nationwide study using the US National Cancer Database 53. It reported on a total of 2753 patients with newly-diagnosis melanoma brain metastases whether with (60%) or without (40%) concurrent extra-cranial metastases. Median overall survival was as short as 1.8 months in patient receiving no form of active anti-cancer treatment. Median overall survival in patients treated with an immunotherapy-containing regimen versus those treated without immunotherapy was 12.4 months and 5.2 months respectively. The four-year overall survival rate was 28.1% with immunotherapy and 11.1% without immunotherapy.

The main reasons for exclusion were due to single arm (non-comparative) studies, wrong intervention (i.e. not immunotherapy), no survival data was presented, wrong comparison, review articles, case series, duplicates and no full text available. Full details are described in Figure 1 (PRISMA diagram).

All included studies were retrospective studies, the treatment would have been selected by the clinician due to various clinical reasons and factors. For example, patients who did not receive immunotherapy with radiotherapy could be due to their poor baseline performance status, hence the lower overall survival. There is high risk of selection bias with studies that are not randomised controlled trials, but unfortunately they are the only types of comparative studies available in the contemporary era. Most studies were data extracted from cancer data base or hospital data base. There was no loss to follow up, therefore the risk of incomplete outcome data is low. None of the studies would have published a study protocol, given they are all observational. However there are large cohort of patients, with multiple studies reported the outcomes from cancer database, there are also multi-center analyses included. The risk of selection bias is low.

Our key findings were that immunotherapeutic treatment strategies were associated with improved overall survival in patients with melanoma brain metastases compared to those receiving supportive care only and that the addition of immunotherapy to brain radiation therapy led to improved overall survival compared with radiotherapy alone.

### Meta-analysis

**Figure 2:**
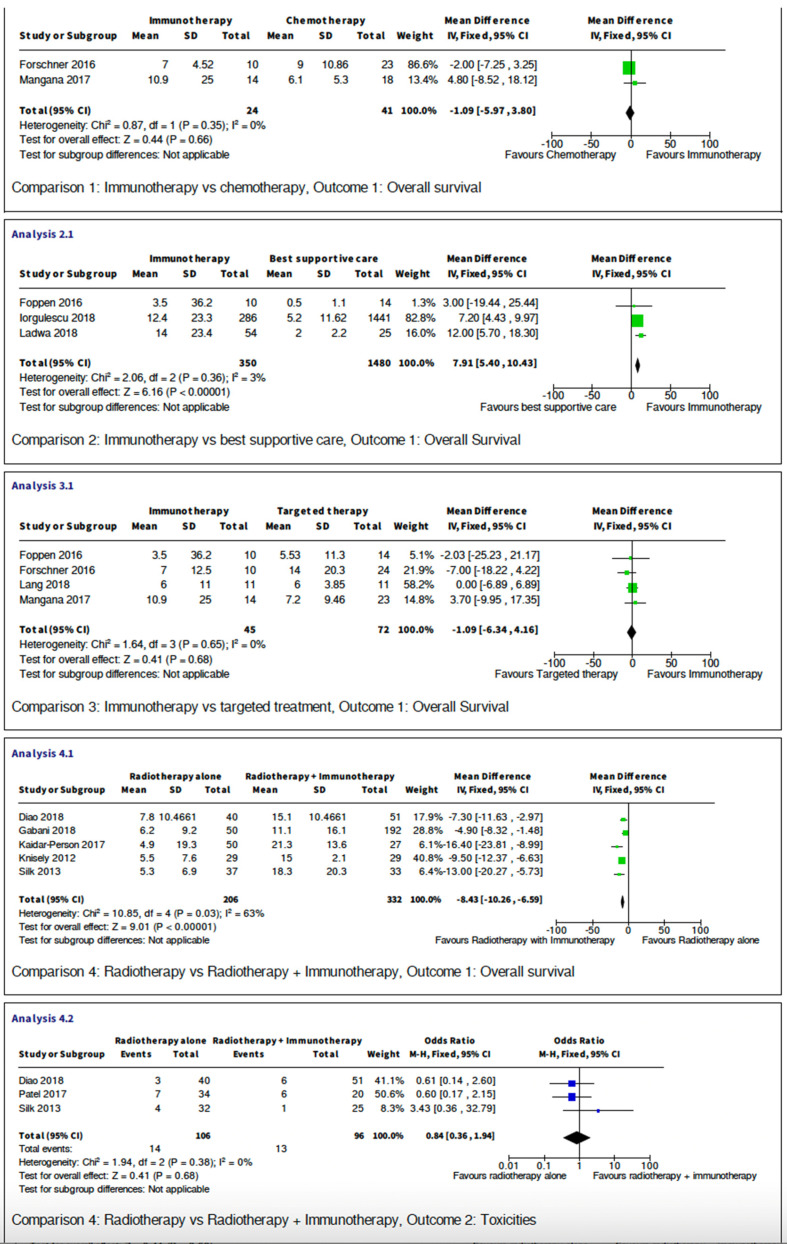
Meta-analysis.

Five studies are retrospective studies comparing immunotherapy with targeted treatment, or best supportive care. Six studies compared outcome between combination of radiotherapy and immunotherapy with radiotherapy alone. The studies all showed heterogeneity and have sufficient data for meta-analysis, which is presented below and in Figure 2.

#### Overall survival

##### Comparison between Immunotherapy and best supportive care

Three retrospective studies have compared 1830 melanoma brain metastasis patients receiving immunotherapy with those who were offered best supportive care only. The analysis has shown that immunotherapy provided a statistically significant increase in overall survival when given to patients compared with best supportive care (p<0.00001). The mean difference is 7.91 with 95% confidence interval of 5.4 to 10.43. I2 test has shown low variability amongst the included studies.

##### Comparison between immunotherapy and cytotoxic chemotherapy

There were only two studies with a direct comparison between immunotherapy and cytotoxic chemotherapy. A total of 65 patients were studied with an odds ratio of -1.09 (95%CI -5.97 to 3.8). Both individual studies 95% confidence interval crossed the null effect line, and when combined this meta-analysis has shown no statistically significant diffence in terms of overall survival (p=0.35). I2 test= 0 indicating low variability between studies.

##### Comparison between immunotherapy and targeted therapy

There were also studies comparing 117 MBM patients receiving immunotherapy with those who received BRAF pathway inhibitors (i.e. RAF +- MEK inhibitors). The mean difference of -1.09 (95% CI -6.34 to 4.16) demonstrate that the 95% CI crosses the line of null effect, suggesting there is not a statistically significant difference in overall survival when comparing patients who received either immunotherapy or targeted therapy. This conclusion is further confirmed by Figure 2 when considering the individuals studies on the forest plot can all be seen to be crossing the null effect line. The heterogeneity of this meta-analysis has shown no variability (I2=0) allowing strong conclusions to be drawn from this data.

##### Comparison between radiotherapy alone with radiotherapy combined with immunotherapy

Finally, in terms of efficacy, there are five studies comparing total of 538 patients receiving only radiotherapy (either stereotactic radio-surgery or whole-brain radiotherapy) with patients that received radiotherapy combined with immunotherapy. The mean difference is -8.43 (95% CI -10.26 to -6.59, p<0.0001) crossing the line of null effect, this suggests that addition of immunotherapy to concurrent radiotherapy is associated with a statistically significant increase in overall survival. However, the heterogeneity of this meta-analysis has shown no variability (I2=63).

#### Toxicities

##### Comparison between radiotherapy alone and radiotherapy and immunotherapy

This meta-analysis compared reported toxicity events between three studies with a total of 202 participants. The reported toxicities are mainly brain radio-necrosis or intra-cranial oedema and intra-cranial haemorrhage. There are no statistically significance differences between the two groups with the 95% confidence interval crossing the null effect line. Odds ratio is 0.84 (95% CI 0.36 to 1.94, p=0.68) with no variability in I2 test. Therefore, this analysis provides evidence that the addition of immunotherapy to radiotherapy treatment does not cause an increase in toxicities.

Our key finding that survival outcomes are superior when melanoma brain metastases patients are treated with immunotherapy rather than supportive care only is entirely in keeping with the small-scale prospective clinical trial data that shows that immunotherapy, in particular anti-PD-1 therapy or combined anti CTLA-4 and anti-PD-1 therapy, is a highly effective treatment options in those with small, asymptomatic brain lesions and may offer some degree of palliative benefit in those with larger, symptomatic and steroid-requiring brain metastases, as discussed below.

The findings of Tawbi are highly pertinent to daily clinical practice in this setting 54. Treatment of 18 symptomatic patients with combination immunotherapy using ipilimumab and concurrent nivolumab led to a median overall survival of 8.9 months and despite a very short median progression-free survival of 1.2 months, two-thirds (66%) of patients were alive at 6 months after treatment initiation. Just under a quarter of patients (22.4%) achieved a radiologic response within the brain metastases. In terms of interpreting the significance of these findings and their generalisability to routine practice, the size of the largest brain metastasis was limited to 3cm, only 7 of 18 patients had 3 or more lesions, only 2 patients had received brain stereotactic radiotherapy before immunotherapy and the maximum permitted corticosteroid dosage was 4mg daily dexamethasone (or equivalent).

Since not all patients with advanced melanoma have sufficient fitness and performance status to safely receive and tolerate combination immunotherapy, the findings of Long *et al.* 55 are also important. In a prospective Phase II study, it was found that in patients with symptomatic brain metastases who had failed local brain therapies or had leptomeningeal metastatic disease, the intra-cranial response rate was low at 6% with nivolumab monotherapy, and median overall survival was as short as 5.1 months. In an earlier study, Margolin *et al.* 56 found that in 21 patients with symptomatic melanoma brain metastases treated with 4 cycles of ipilimumab monotherapy, intra-cranial disease control was achieved in 10% of patients. However, in these patients most of whom were requiring corticosteroids, median overall survival was short at 3.7 months with median progression-free survival of 1.3 months. 1 and 2-year overall survival rates were 19 and 10% respectively, however, suggesting that a small group of treated patients could achieve durable disease control.

There were 4 prospectively-enrolling clinical trials including patients with asymptomatic melanoma brain metastases in patients not requiring corticosteroids for control of neurologic deficits. The role of anti-PD-1 monotherapy was evaluated in this patient population by Kluger *et al.* 57 in a single-arm prospective Phase II study of pembrolizumab in patients with small (5-20mm) asymptomatic brain metastases. An intra-cranial objective response rate of 26% was achieved. Although median progression-free survival was very short at 2 months, the median overall survival was a respectable 17 months and 1 and 2-year overall survival rates were 60% and 48% respectively.

Combination immunotherapy with ipilimumab-nivolumab in patients with asymptomatic melanoma brain metastases was evaluated by Tawbi *et al.* 54. In 101 patients, median overall survival was 46 months with a 72% 3-year overall survival rate and 39 months median intracranial progression free survival. The intra-cranial objective response rate was 53%.

In a similar vein, Long *et al.* 31 studied the role of ipilimumab-nivolumab in patients with asymptomatic melanoma brain metastases. The rates of intracranial progression-free survival at 1 and 2 years were 49% and 49% with ipilimumab-nivolumab and 20% and 15% with nivolumab and 1 and 2 year overall survival rates were 63 and 63% with ipilimumab-nivolumab and 60 and 51% with nivolumab. Intracranial objective response was achieved in 51% with ipilimumab-nivolumab and 20% with nivolumab.

An earlier study by Di Giacomo *et al.* 58 found a median overall survival of 13.4 months with a 1-year overall survival probability of 54% and a 50% chance of intracranial disease control using ipilimumab and concurrent fotemustine chemotherapy, although median intracranial progression-free survival was short at 3 months.

**Figure 3:**
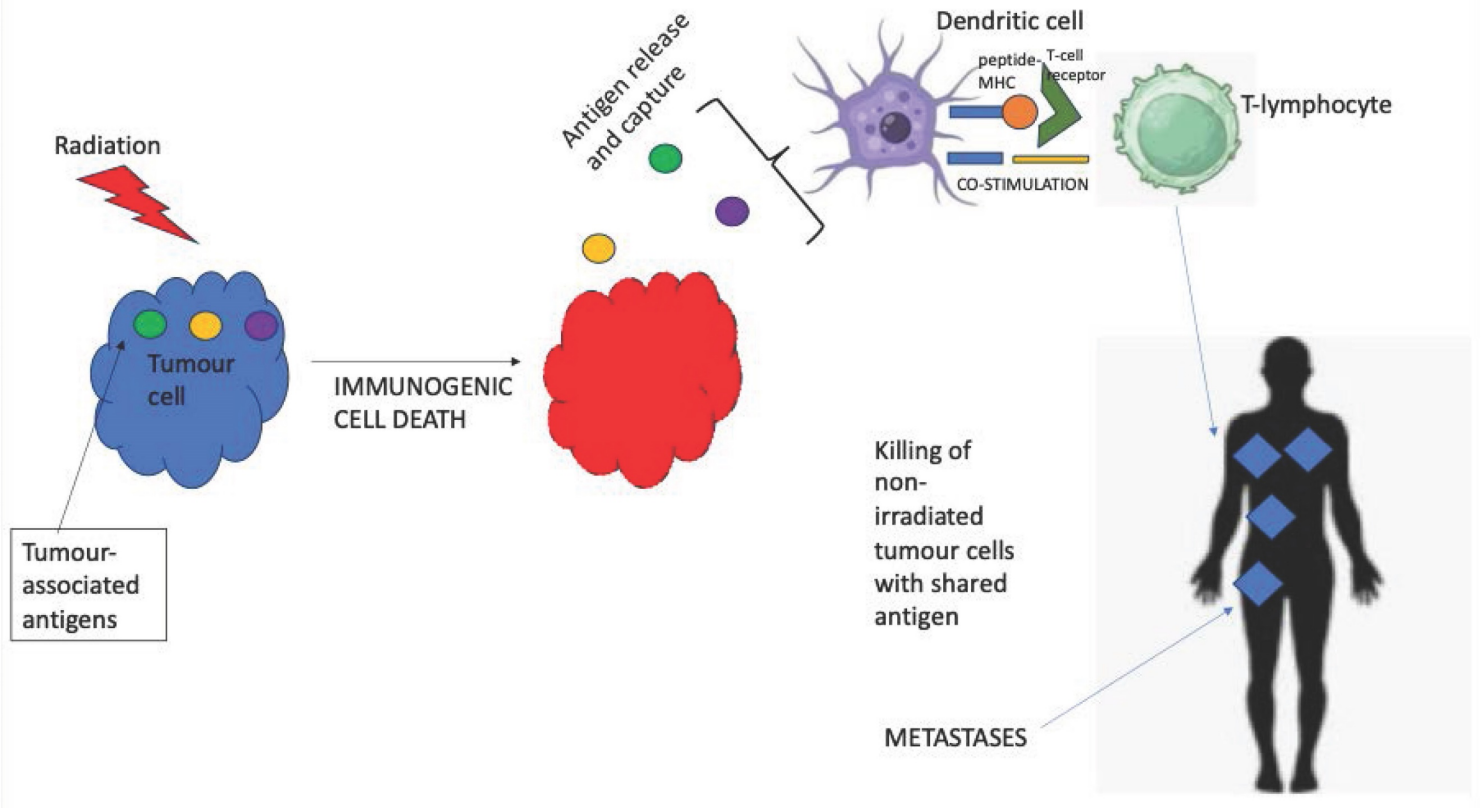
Visual representation of the 'abscopal' effect.

Broadly speaking, our finding that overall survival was superior with stereotactic radiotherapy alongside concurrent immunotherapy for patients with melanoma brain metastases is concordant with the results of other reviews and meta-analyses such as that of Tan *et al.* 59 and Lancellotta *et al.* 60. This suggests that the biological concept of 'abscopal' effect (Figure 3) may be a clinical reality with localised radiotherapy leading to immunogenic cell death of the melanoma cells, consequent tumour antigen release and immune priming and hence re-invigorated immune response to checkpoint inhibition 61. Importantly, the rates of clinically-significant neurological toxicity were low even with concurrent radiation and immunotherapy.

Interestingly, a retrospective cohort study of 105 melanoma brain metastases patients treated with concurrent systemic therapy and localised brain radiotherapy found that although high (greater than two times the upper limit of normal) serum LDH levels were an adverse prognostic factor for overall survival in those receiving BRAF-targeted therapy, this was not the case for those receiving immune checkpoint inhibitors 62.

In terms of our finding that there seemed to be equivalence of overall survival between immunotherapy and molecularly-targeted therapy (RAF and MEK inhibitors) this is slightly divergent from the current consensus view that long-term disease control is most likely to be achieved with immunotherapy rather than targeted treatment. However, most of the patients treated with immunotherapy in our review received ipilimumab monotherapy and many received combined RAF-MEK inhibitors which may explain this, and patient numbers were small. In this regard, it is also important to note that clinical trials (e.g. STARBOARD NCT04657991) are ongoing to evaluate triplet-therapy (anti PD-1 therapy with concurrent RAF and MEK inhibitors) as first-line treatment for advanced BRAF mutant melanoma, so in the future the comparison between targeted therapy and immunotherapy as single modalities of treatment may become less relevant.

Our finding, based again on a small number of patients, that there was no significant difference in overall survival comparing immunotherapy to chemotherapy, is not entirely divergent from the results of 2 randomised controlled Phase III clinical trials - Keynote-002 where median overall survival for Stage IV melanoma patients treated with pembrolizumab was numerically but not statistically significantly higher than that of those treated with investigators' choice of chemotherapy 15, and Checkmate-037 where nivolumab treatment was not associated with statistically significantly superior overall survival compared to chemotherapy 63. In both these randomised trials, the findings were attributed to extensive cross-over from chemotherapy to immunotherapy at the time of progression and imbalances in baseline prognostic factors between the treatment groups that favoured the chemotherapy groups. On the other hand, the Checkmate-066 study found significantly improved long-term overall survival with nivolumab versus dacarbazine chemotherapy with 5-year overall survival of 38% with immunotherapy and 17% with chemotherapy 64. In contemporary melanoma oncology practice, chemotherapy plays a very limited role in advanced melanoma although it may offer transient palliation and symptom control and may have a greater role in mucosal melanoma 65.

## Conclusions

High-quality, prospective clinical studies suggest that patients with melanoma brain metastases can be effectively treated with systemic immunotherapy in particular immune checkpoint inhibitors such as ipilimumab and nivolumab; especially when the brain lesions are small, asymptomatic and not requiring steroids to control peri-lesional oedema. In fact, the efficacy of combined anti CTLA-4 and PD-1 treatment is equivalent for those with and without asymptomatic brain metastases. Very recently, however, the combination of nivolumab with the anti-LAG3 antibody relatlimab has emerged as a potential front-line therapeutic option for advanced melanoma with a more favourable toxicity profile than ipilimumab-nivolumab and significant biological activity in high-risk subgroups 66. The brain activity of this regimen remains to be demonstrated, however. Our review suggests that immunotherapy alongside stereotactic radiotherapy leads to improves overall survival compared with radiotherapy alone and that overall survival is improved with immunotherapeutic approaches versus supportive care only. It remains critically important to perform regular neuro-imaging for patients with stage III and IV malignant melanoma, ideally using magnetic resonance imaging, in order that any dissemination of the cancer to the central nervous system can be detected early prior to symptoms developing to allow for successful immunotherapy, and also at a stage where stereotactic radio-surgical approaches are feasible in view of melanoma's extreme refractoriness to conventionally delivered whole-brain radiotherapy. Patients must be counselled and educated to report any neurologic symptoms to their physician promptly. For patients with the BRAF V600 mutation, our findings suggest that combined RAF-MEK inhibitors should be considered as an upfront systemic therapy option, although we note the findings of the DREAMSeq trial 67 that suggest the preferred sequence is immunotherapy followed by molecularly-targeted therapy. The DREAMSeq trial, however, only included 4 (of 265) patients with very small and asymptomatic brain metastases. The other pertinent trial was the SECOMBIT study where the 3-year overall survival rate was numerically higher with an immunotherapy first approach, although no patients with 'active' brain metastases were included 68. Regardless of the magnitude of overall survival benefit, it is clear that combined RAF and MEK inhibitors can achieve rapid and deep intra-cranial responses which are associated with improved neurologic status in many patients. There is an informative retrospective database-based study, however, which indicated that the incidence of newly-developed brain metastasis was higher in patients treated with 1^st^ line targeted therapy compared with immunotherapy for patients with either Stage III resected melanoma or those with Stage IV disease (without initial brain metastases). In a retrospective cohort of 683 BRAF V600 mutated patients, treated over an 8-year period (2011-2019), it was found that the incidence of brain metastasis was approximately double with initial targeted therapy versus immunotherapy (31 versus 16%) and median brain metastases-free survival was 11 months with targeted therapy compared with 42 months with immunotherapy. Therefore, this study suggests that immunotherapy is more effective than targeted therapy in preventing or delaying the occurrence of brain metastases from melanoma, in both adjuvant and metastatic contexts 69.

It is also important to note that many of the earlier studies included in our review and meta-analysis used varying doses of ipilimumab (monotherapy) - ranging from 1mg/kg to 10mg/kg and this heterogeneity may also have influenced the clinical outcomes. There is evidence to suggest that use of the 10mg/kg dose of ipilimumab may improve overall survival to a greater extent than the 3mg/kg dose in patients with asymptomatic brain metastases 70.

Although immunotherapy as a single modality of treatment can be effective in patients who are asymptomatic neurologically, it is vitally important to begin to understand the potentially synergistic interactions between localised therapeutic modalities such as stereotactic radiotherapy and immune-based treatment in terms of both safety and efficacy and how to exploit such synergism for maximal patient benefit. Novel study designs or research paradigms may need to be considered, especially in view of the very slow recruitment in the PERM trial of pembrolizumab with or without stereotactic ablative radiotherapy that was terminated early (NCT02562625). Research is needed to establish the importance of the temporal relationship between SRS and immunotherapy, and it appears that to gain maximal therapeutic benefit and exploit the synergism between radiotherapy-driven tumour antigen release (and immune priming) and immune checkpoint inhibition, concurrent treatment is superior to non-concurrent approaches (immunotherapy up to two weeks before or 2 weeks after radiotherapy) 71. Patients with leptomeningeal metastases are rare and poorly studied, and this patient population deserves more attention in prospective studies. For patients with symptomatic brain metastases, approaches to reducing peri-lesional oedema without using corticosteroids require active investigation. The role of intrathecal immunotherapy needs to be determined for patients with lepto-meningeal dissemination. In terms of the subgroup of BRAF mutant patients with Stage IVd melanoma, whether immunotherapy should be used second-line after initial RAF-MEK inhibitor treatment, or in the first-line setting or perhaps concurrently with targeted therapy, remains uncertain, in view of the very small number of patients with brain metastases in the DREAMSeq and SECOMBIT trials. A key research question, especially in view of the increasingly widespread adoption of adjuvant systemic immunotherapy for patients with Stage II and III melanoma is whether adjuvant immunotherapy prevents and/or delayed the development of CNS metastases. Although our review suggests anti CTLA-4 and PD-1 based immunotherapy can be effective in the brain, the role of other forms of immunotherapy such as adoptive T-cell therapy and redirected T-cell therapy 72 also need to be explored. The key potential advantages and disadvantages of immunotherapy for intra-cranially metastatic melanoma are summarised in Table 1. It remains important to understand the determinants of quality-of-life, especially in terms of neurological disability, in these patients with melanoma brain metastases undergoing systemic and local treatments.

## Figures and Tables

**Table 1:** Key advantages and disadvantages of immunotherapy of intra-cranial melanoma metastases.

Advantages	Disadvantages
The brain is not an immune-privileged site and activated T-cells may be able to target intra-cranial metastases	Immune checkpoint inhibitors, having no direct effect on the tumour cells themselves, can be slow in achieving therapeutic effect which could make treating imminently life-threatening metastatic disease within the brain a challenge
Immunotherapy may be able to drive highly specific, durable memory T-cell responses to tumour antigens potentially allowing long-term control of brain metastases	There is a risk of immunotherapy-related exacerbated peri-lesional oedema and haemorrhage in brain metastases from melanoma and a risk of radio-necrosis when concurrent stereotactic radiotherapy is used
Immunotherapy can be employed for all melanoma patients irrespective of the molecular profile of the melanoma	Steroids, often required for symptomatic brain metastases, may suppress the efficacy of immunotherapy
There is a strong rationale for synergism between localised radiotherapy and immunotherapy via the abscopal effect, increasingly important with the widespread adoption of stereotactic radiation.	
